# Cost effectiveness analysis of total laparoscopic hysterectomy versus total abdominal hysterectomy for uterine fibroids in Western China: a societal perspective

**DOI:** 10.1186/s12913-022-07644-9

**Published:** 2022-02-24

**Authors:** Jinjuan Yang, Xiaojing Fan, Jianmin Gao, Dan Li, Yongjian Xu, Gang Chen

**Affiliations:** 1 School of Public Health, Xi’an Jiaotong University Health Science Center, Xi’an, 710061 People’s Republic of China; 2 School of Public Policy and Administration, Xi’an Jiaotong University, Xi’an, 710049 People’s Republic of China; 3 School of Public Management, Northwest University School, Xi’an, 710127 People’s Republic of China; 4 Monash Business School, Monash University, Clayton, VIC 3145 Australia

**Keywords:** Uterine fibroids, Incremental cost effectiveness ratio, Total laparoscopic hysterectomy, Total abdominal hysterectomy

## Abstract

**Background:**

As a common female pelvic tumor, uterine fibroids remain the leading cause for hysterectomy in China. Hysterectomy provides a good surgical treatment of uterine fibroids, and it guarantees the removal of all uterine fibroids without lower risk of recurrence. This study compares the cost effectiveness of total laparoscopic hysterectomy (TLH) versus total abdominal hysterectomy (TAH) for women with uterine fibroids from a societal perspective.

**Methods:**

An economic analysis was conducted in 392 patients (TLH *n* = 75; TAH *n* = 317), including all relevant costs over a 12-month time horizon. Primary outcome was major surgical complications; secondary outcomes were postoperative discomfort symptoms and time of return to normal activities. Clinical, outcomes and costs data were collected from medical records, telephone survey and financial information system. Generalized linear models were used to assess costs and outcomes differences between the two groups. Incremental cost effectiveness ratio (ICER) was used to estimate the cost effectiveness.

**Results:**

Mean direct costs were $2,925.71 for TLH, $2,436.24 for TAH, respectively. Mean indirect costs were $1,133.22 for TLH, $1,394.85 for TAH, respectively. Incremental societal costs were $256.86 (95%CI: 249.03–264.69). Mean differences in outcome were: 4.53% (95%CI: 4.35–4.71) for major surgical complications; 6.75% (95%CI: 6.45–7.05) for postoperative discomfort symptoms; 1.27 (95%CI: 1.23–1.30) weeks for time to return to normal activities. ICER of TLH was $5,669.16 (95%CI: 5,384.76–5,955.56) per complication averted, $3,801.54 (95%CI: 3,634.81–3,968.28) per postoperative discomfort symptoms averted and $202.96 (95%CI: 194.97–210.95) per week saved to return to normal activities.

**Conclusions:**

TLH is cost effective compared with TAH in preventing additional complications based on our estimated conservative threshold in China. The findings provide useful information for researchers to conduct further cost effectiveness analysis based on prospective study which can provide stronger and more evidence, in China. In addition, the data may be useful for Chinese health care policy-makers and medical insurance payers to make related health care decisions.

## Introduction

Uterine fibroids are the most common female pelvic tumor, causing lots of health risks of women [1–3]. It is reported that 60% of women of reproductive age are attacked with uterine fibroids, and 80% of women develop the disease during their lifetime [4]. While most fibroids are asymptomatic, they are clinically apparent in up to 30–40% of women aged 40 and older and up to 25% of women of all ages [1, 5]. Hysterectomy provides a good surgical treatment of uterine fibroids, and it guarantees the removal of all uterine fibroids without lower risk of recurrence [6]. Hysterectomy has been the major surgical treatment of uterine fibroids, approximately 75% of all fibroid treatments [3, 4]. Hysterectomy is the most common nonpregnancy-related gynecologic surgical procedure performed worldwide, with rates of 3.62 per 1,000 women in Germany [7], 3.12 per 1,000 women in Australia [8], 5.1 per 1,000 in 2004 in the United States and 17 per 1,000 women in India [9] and uterine fibroids are the leading cause of hysterectomy [10, 11]. In the United States, it is estimated that annual direct costs of hysterectomy were $0.78 to $3.5 billion and lost work costs ranged from $0.55 to $9.35 billion annually [12]. Health care expenses and related indirect costs, such as the losses of monetary income due to disability and time out of work, cause a significant societal and economic burden. However, there is a lack of information on the incidence of uterine leiomyoma with national database in China. Gu et al. reported that 147,966 patients with leiomyomas were performed by hysterectomy in hospitals (excluding military hospitals) in mainland China in 2010 [13].

To date, various hysterectomy procedures with minimized invasiveness have been developed [14–21]. The main approaches for hysterectomies are abdominal, vaginal, laparoscopic, and robotic assistance [22]. Extensive studies compared the advantages of various hysterectomies. Most studies have shown that laparoscopic hysterectomy requires smaller incisions, less intraoperative blood loss, shorter length-of-hospital stay (LOS), faster recovery, better short-term quality of life (QOL) and quicker return to activity in daily life or work, as compared with abdominal hysterectomy or vaginal hysterectomy [23–27]. However, according to a multicentre randomised trial that evaluated the outcomes and cost effectiveness of abdominal hysterectomy, vaginal hysterectomy and laparoscopic hysterectomy, the results showed that laparoscopic hysterectomy was associated with a significantly higher rate of major complications and required longer operative times [28]. Aarts et al. evaluated 47 randomised controlled trials and found that laparoscopic hysterectomy had a greater risk of urinary tract injury [22].

Cost effectiveness analysis can provide an important tool in determining which procedure is cost effective for patients, payers and policy makers. Findings on the cost effectiveness of various hysterectomies are controversial. Rutstein et al. reported that laparoscopic hysterectomy was cost effective compared with abdominal hysterectomy or vaginal hysterectomy, when considering total direct hospital costs, complications, and morbidity [29]. Graves et al. found that TLH was cost effective procedure compared to TAH, for early endometrial cancer, when measured by quality-adjusted life years (QALYs) [30]. On the other hand, Sculpher et al. compared one-year costs and QALYs of abdominal hysterectomy, vaginal hysterectomy and laparoscopic hysterectomy [31] and Garry et al. performed a multicentre randomised trial comparing abdominal hysterectomy, vaginal hysterectomy and laparoscopic hysterectomy [28]. Both studies found that the cost effectiveness of laparoscopic hysterectomy in the comparison with abdominal hysterectomy is finely balanced, and laparoscopic hysterectomy was not cost effective compared to vaginal hysterectomy. However, both studies estimated the costs of different hysterectomies from a health service perspective, and did not analyze the productivity savings in monetary terms. When the productivity losses of patients and caregivers were considered, laparoscopic-assisted vaginal hysterectomy is found to be the most cost-effective procedure [23].

Due to disparities in demographic factors, economic, medical level and health care system, a diversity of costs associated with hysterectomy was found among several countries [27] even among various regions in the United States [32]. Previous research in mainland China compared the clinical effects of different hysterectomies [20, 33–37], or the costs during the hospitalization [13, 38, 39]. There is a relative absence of data on social costs including healthcare costs and productivity losses in mainland China. To date, few studies have performed a cost effectiveness analysis of various hysterectomy procedures in China, from a societal perspective. To our best knowledge, only Leng et al. compared costs, health care utilization and QOL of TAH, laparoscopic hysterectomy and TVH, but they made the comparison only in a short 28-day time horizon [38]. A longer, broader, societal perspective is therefore needed to estimate the cost effectiveness of different hysterectomy procedures in mainland China. Therefore, our study aims to evaluate the cost effectiveness of TLH versus TAH for women with uterine fibroids in Western China, from a societal perspective, over a 12-month time horizon.

## Methods

### Data

**Fig. 1:**
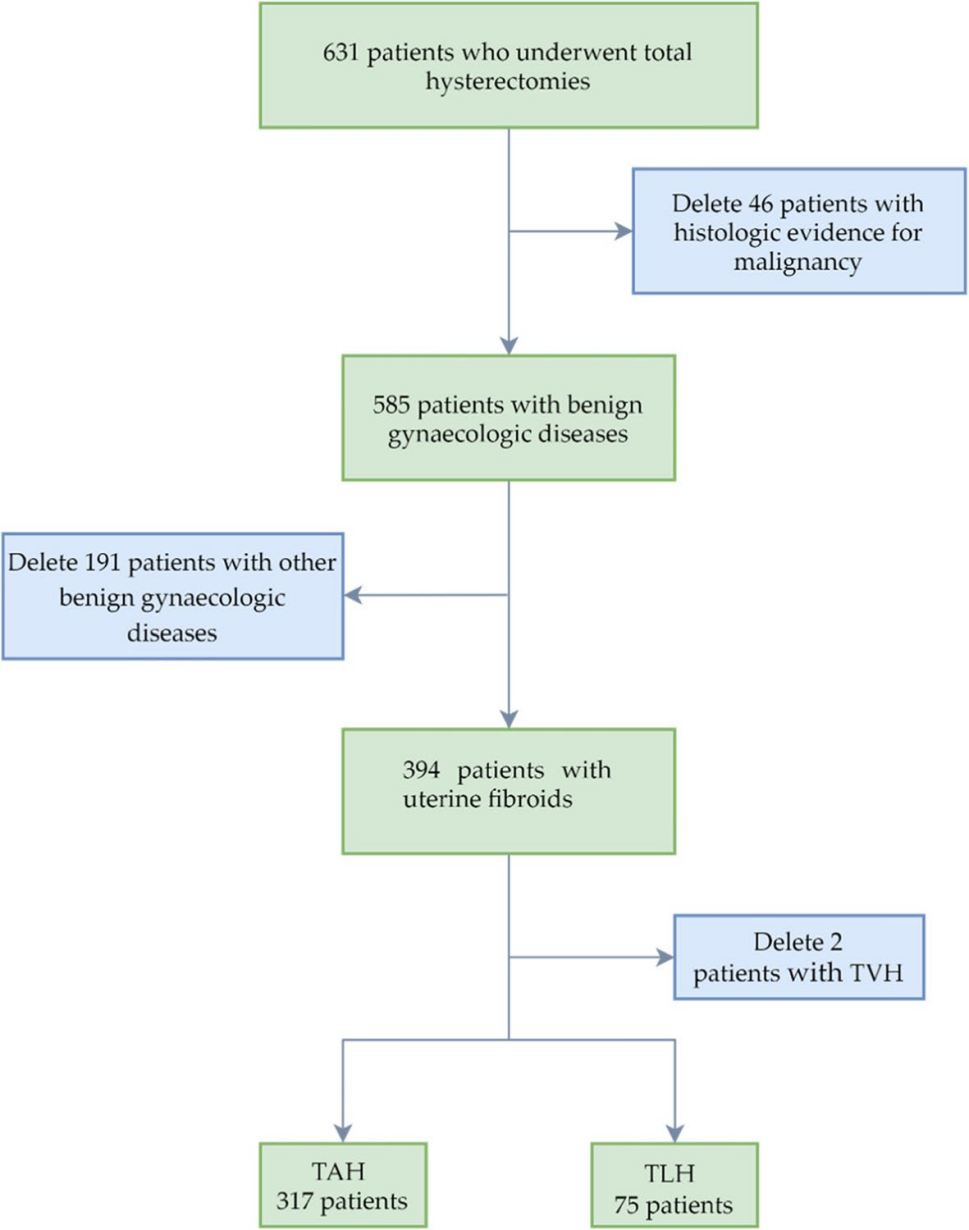
Flowchart on the sample selecting process of this study.

A retrospective observational study was performed of patients who underwent total hysterectomy for uterine fibroids by TAH and TLH, between January 1, 2011 and December 31, 2012, at the First Affiliated Hospital of Xi'an Jiaotong University (FHXJTU). Patients received surgical treatment according to an International Classification of Diseases, ninth revision, Clinical Modification codes: 68.4 01 for TAH and 68.4 05 for TLH, at FHXJTU, during 2011 to 2012. All the operating surgeons had more than 10-year of experience as gynaecologists, and laparoscopic surgeons were well-trained and experienced. Patients included were diagnosed with uterine fibroids. We excluded patients with histologic evidence for malignancy or patients with other benign gynaecologic diseases. During the study period, two patients with uterine fibroids chose TVH. Rather, the majority of TVH patients had the procedure because of a uterine prolapse. The indication for TAH or TLH was: a) symptomatic uterine fibroids, including heavy menstrual bleeding, pelvic pressure and pain; b) no desire to bear children, and c) the patient's personal preference and beliefs or attitudes towards TAH or TLH. There were no significant differences in the indications between the two procedures, TAH and TLH. The study compared two procedures of TAH and TLH. The final study sample (*n* = 392) was composed of 317 patients who underwent TAH and 75 patients who underwent TLH. The flowchart on the sample selecting process of this study is shown in Fig. 1.

### Measurement

#### Socio-demographic and clinical characteristics

Socio-demographic characteristics include each patient's age, insurance status, employment status and living location. The clinical characteristics includes body mass index (BMI), number of births, previous delivery, menopause, type and size of fibroid, previous abdominal operation, contraceptive ring, disease severity on admission, and comorbidity. Comorbidities includes hypertension, diabetes, anemia, respiratory tract infection, asthma, hepatitis, ovarian cyst, cervicitis, pelvic inflammatory disease, bladder distention, uterine prolapse, hyperthyroidism, hypothyroidism, congenital heart disease. Surgical procedures data includes type of anaesthesia, time in operating room, estimated blood loss (EBL) during surgery, received blood transfusion, secondary procedures, uterine volume. Other clinical data includes antibiotic usage, rate of major surgical complications, pre-operative and post-operative length of stay (LOS) and discomfort symptoms. Socio-demographic and clinical characteristics were collected from the patients' medical records.

#### Effectiveness

The primary measure of effectiveness was major surgical complications, which include both intraoperative complications and postoperative complications. Intraoperative complications were defined as ureteral injury, bladder injury, or EBL of more than 1,000 ml. Postoperative complications were defined as fever which was defined as an oral temperature of 38 °C or greater, on two successive occasions 4 h apart, excluding the first 24 operative hours [40], incision dehiscence, secondary suture, intestinal obstruction and hospitalization of more than 30 days. Period of hospitalization was chosen rather than 30-day readmission because no study patient was readmitted to a hospital within 30 days [26, 30, 41].

Rate of discomfort symptoms, time of return to normal activity (defined as able to perform most activities at 90% of preoperative levels), were used as secondary measures of effectiveness. After the hysterectomy, discomfort symptoms include wound pains, vaginal pains, gas pains, bowel symptoms, bloating, red vaginal bleeding, trouble passing urine or stool, granulation tissue, etc. Outcome data of measures of effectiveness were obtained from the patients' medical records and from interviews with the patients during their postoperative visits or by telephone survey.

#### Costs

To evaluate the economic consequences of two procedures from a societal perspective, we assessed the direct costs (including direct medical costs and direct non-medical costs), and productivity losses of both patients and caregivers [42]. Direct medical costs refer to all medical expenses incurred during the hospitalization, surgery, drugs (both western and traditional Chinese medicine), treatment, investigations (e.g., X-ray, CT, electrocardiogram, blood tests, et al.), nursing care, consultations, blood transfusions and ward stay. We define the medical cost as the payments by the patient and insurance scheme. Direct non-medical costs refer to the patient's transportation costs when travelling to the hospital. Indirect costs refer to the value of the productivity losses by both the patients and the caregivers after the operation. Indirect costs in this study were calculated by multiplying the number of weeks absent from work by the patient's estimated weekly wage rate. In the case of retired or nonworking patients, costs of productivity loss could not be assessed using this method. However, it is incorrect to neglect the value of reduced time for leisure activities, and unpaid work (e.g., housekeeping, voluntary community work) [43]. In terms of methodology how to value the loss of time of retired and nonworking patients, some published studies used a proxy good method to value the reduction of time of unpaid services, leisure activities, and voluntary community work, by using equivalent market price for unpaid productivity (i.e., market wage rate of a housekeeper) [44, 45]. Therefore, indirect costs for those retired and unemployed patients, we calculated by multiplying the weeks to normal activity with the market wage rate of housekeeping (i.e., $56.6 per week in 2011) [46]. A sensitivity analysis with a range of ± 20% of the market wage rate of a housekeeper, was performed to estimate productivity losses of retired and nonworking patients. All costs were adjusted for inflation to 2020 values using 3% discount rate and reported in 2020 U.S. dollars ($1 = ¥ 6.900) [47]. Medical costs data was collected from the financial information system of FHXJTU, whilst direct non-medical costs and indirect costs data were collected through interviews with the patients during their postoperative visits or by telephone survey over a 12-month period.

#### Cost effectiveness analysis

The ICER was used to estimate the cost effectiveness of TLH versus TAH for women with uterine fibroids. The ICER was calculated as the incremental cost between TLH and TAH, divided by the difference in their effect.

Multi-variable adjustments of cost and effect were performed using general liner models (GLM) to adjust for the potential differences on preoperative socio-demographic and clinical characteristics between two patient groups. GLM with a log-link function and gamma distribution, was used to adjust the costs, LOS and time to normal actives, whilst rate of major surgical complications and rate of discomfort symptoms, were adjusted by GLM with a logit-link function and binomial distribution. Bootstrapping on patients' adjusted costs and effects across 1,000 replicates was performed to allow for robust assessment of uncertainty of costs, effects and cost effectiveness. Ninety-five percent confident intervals (95% CIs) were calculated for the ICERs using these replicates.

To date, there is no universally accepted threshold of the acceptable cost for the effect measures used as major surgical complications. When the outcome measure is preference-based utility score and the ICER is expressed as cost per QALY gain or cost per disability-adjusted life-year avoided, the World Health Organization has suggested a cost effectiveness threshold of three times the national annual gross domestic product (GDP) per capita [48]. A previous systematic review suggested an acceptable ICER would not exceed a conservative threshold of US$20,000 per the reduction of one additional major complication [24]. The $20,000 figure was one half of the threshold of cost per QALY adopted by the National Institute for Health and Care Excellence in the UK at that time. According to this method, a threshold of cost to prevent one additional complication is about one and a half times the local GDP per capita. Considering the GDP per capita in China was US$5,618 in 2011 (inflated to 2020, $7,033) [49] we estimate the threshold of cost to prevent one additional complication to be $10,995 in our study.

#### Ethics statement

Ethics approval and consent for the study was approved by the Ethics Committee of the Xi'an Jiaotong University Health Science Center (approval date: 30/6/2014). Prior to the telephone interview, verbal consent was obtained from all subjects involved in the study. All patient information was anonymised and deidentified prior to analysis.

### Statistical analysis

For the normally distributed continuous variables, analysis of variance (ANOVA) or t test was used, whilst for categorical data, chi-squared tests or Fisher exact tests were conducted to study statistical significance between the two patient groups. Non-normally distributed variables (based on the Shapiro–Wilk test) were reported as the median with the interquartiles (i.e., Q1-Q3) or mean ± the standard deviation (e.g. LOS and costs of distinct surgical types) and were compared by using the nonparametric Mann–Whitney U test.

The multiple imputation by chained equations (MICE) method [50] was used to deal with missing data in three variables—days back to normal activity (*n* = 89), days back to work (*n* = 89), and discomfort symptoms (*n* = 89). Data were missing in 89 cases (TAH: 73, TLH: 16), out of 392 included patients (TAH: 317, TLH: 75). There was no significant difference in rate of missing values between the two groups (*p* > 0.05). The imputation procedure used an iterative Markov chain Monte Carlo (MCMC) method based on multivariate normal or logistic regression [50] and involved replacing each missing value in the dataset with 20 plausible values that presented the uncertainty. The 20 resultant multiple imputed datasets were then analysed using standard complete-case procedures and the results were then combined using Rubin rules [51].

All statistical tests were 2-sided. *P* value less than 0.05 was considered statistically significant. Analyses were performed using the Stata version 12.0 (StataCorp LP, College Station, Texas, USA) and Microsoft Access (Microsoft Corporation, Redmond, Wash).

## Results

Patients' socio-demographic and clinical characteristics are summarized in Table 1. Mean ± standard deviation (SD) age were 45.88 ± 4.13 years and 45.29 ± 4.48 years for the TLH and TAH patient groups, respectively. Most characteristics of patients are comparable between the two groups. There were no significant differences between the two groups in age, BMI, number of births, previous delivery, menopause, size of fibroid, previous abdominal operation history, whether they used a contraceptive ring, severity on admission, comorbidities, insurance status, employment status or living location (all *p* > 0.05). Patients in the TLH group were significantly more likely to have a single fibroid than multiple fibroids, compared to patients in the TAH group (*p* = 0.002).

**Table 1:** Socio-demographic and clinical characteristics

Variable	TLH (*n* = 75)	TAH (*n* = 317)	P
Age (years)	45.88 ± 4.13	45.29 ± 4.48	0.299
Body Mass Index	23.24 ± 2.03	23.71 ± 2.61	0.084
Number of births			0.802
0	1(1.33%)	10(3.15%)	
1	42(56.00%)	173(54.57%)	
2	24(32.00%)	91(28.71%)	
≥ 3	8(10.67%)	41(13.56%)	
Previous delivery			0.086
None	1(1.33%)	10(3.15%)	
Vaginal	71(94.67%)	270(85.18%)	
Cesarean	3(4.00%)	37(11.67%)	
Menopause			0.669
Yes	3(4.00%)	11(3.47%)	
No	72(96.00%)	301(94.95%)	
Unknown	0(0%)	5(1.58%)	
Type of fibroid			0.002^*^
Single	40(53.33%)	108(34.07%)	
Multiple	35(46.67%)	209(65.93%)	
Size of fibroid			0.142
≥ 5 cm	38(49.33%)	186(58.68%)	
< 5 cm	37(50.67%)	131(41.32%)	
Previous abdominal operation			0.054
Yes	18(24.00%)	113(35.65%)	
No	57(76.00%)	204(64.35%)	
Contraceptive ring			0.131
Yes	2(2.67%)	25(7.96%)	
No	73(97.33%)	289(92.04%)	
Disease severity on admission			0.116
General	75(100.00%)	307(96.85%)	
Severe	0(0.00%)	10(3.15%)	
Comorbidity			0.328
None	36(48.00%)	128(41.84%)	
One	27(36.00%)	116(36.48%)	
≥ Two	12(16.00%)	73(21.68%)	
Insurance status			0.116
NCMS	36(48.00%)	130(41.01%)	
URBMI	31(41.33%)	113(35.65%)	
UEBMI	6(8.00%)	52(16.40%)	
Uninsured	2(2.67%)	22(6.94%)	
Employment status			0.125
Unemployed	7(9.33%)	29(9.15%)	
Employed	64(85.33%)	283(89.27%)	
Retired	4(5.33%)	5(1.58%)	
Living location			0.664
Xi'an City, Shaanxi Province	26(34.67%)	127(40.06%)	
Other cities in Shaanxi Province	46(61.33%)	177(55.84%)	
Other Provinces	3(4.00%)	13(4.10%)	

*Note*, The data are presented as the mean ± *SD* for continuous variables and as number and percentage (%) for categorical variables. The p-value was derived by two-sample t test for continuous variables and by Pearson’s Chi-square or Fisher’s exact test for categorical variables; *NCMS*,.New Cooperative Medical Scheme, *URBMI*, Urban Resident Basic Medical Insurance, *UEBMI*, Urban Employee Basic Medical Insurance. *TAH*, total abdominal hysterectomy, *TLH*, total laparoscopic hysterectomy; ^*^ *p* < 0.05

Table 2 presents the surgical (including both intraoperative and postoperative) characteristics and clinical outcomes. There were significant differences between the two surgical groups with respect to the time spent in the operating room (time in OR) and uterine volume. On average, compared to the TAH group, the TLH group had significantly longer OR time (163 vs. 130 min, *p* < 0.001) and lower uterine volume (360 vs. 448 cm^3^, *p* = 0.026). Both groups reported similar types of anesthesia, blood loss during surgery, blood transfusions, number of antibiotic use and proportion of patients receiving secondary procedures (all *p* > 0.05). Regarding the effectiveness indicators, there were no significant differences between the two groups in rate of major surgical complications (primary effectiveness measure, including ureteral injury, bladder injury, fluid accumulating, EBL during surgery of more than 1000 ml, fever, incision dehiscence, intestinal obstruction, hospitalization of more than 30 days, and secondary suture) or discomfort symptoms (secondary effectiveness measure). Compared to patients who received TAH, patients who received TLH had a shorter length of hospital stay (9.91 days vs 13.00 days, *p* < 0.001), shorter length of pre-operative stay (3.88 days vs 4.74 days, *p* = 0.005), and shorter length of post-operative stay (6.03 days vs 8.26 days, *p* < 0.001).

**Table 2:** Surgical data and clinical outcomes

Variable	TLH (*n* = 75) ^1^	TAH (*n* = 317)	*P*
Anesthesia			0.252
General anesthesia	75(100.00%)	303(95.58%)	
Combined spinal epidural anesthesia	0(0.00%)	13(4.10%)	
Epidural anesthesia	0(0.00%)	1(0.32%)	
Time in operating room (minutes)	165(140–205)	130(110–150)	< 0.001^*^
Blood loss for surgery (ml)	200(100–300)	100(80–200)	0.139
Received blood transfusion			0.782
Yes	15(20.00%)	59(18.61%)	
No	60(80%)	258(81.39%)	
Uterine volume (cm3)	360(224–607.75)	448(280–780)	0.026^*^
Antibiotic types			0.162
One	20(26.66%)	55(17.35%)	
Two	53(70.67%)	246(77.60%)	
Three	2(2.67%)	16(5.05%)	
Secondary Procedures (% of patients)			0.895
Yes	13(17.33%)	57(17.98%)	
No	62(82.67%)	260(82.02%)	
Rate of major surgical complications	4(5.33%)	31(9.78%)	0.161
Ureteral injury	1(1.33%)	0(0%)	0.191
Bladder injury	0(0%)	1(0.32%)	0.809
Blood Loss for Surgery more than 1000 ml	0(0%)	4(1.26%)	0.426
Fever	2(2.67%)	23(7.26%)	0.109
Incision dehiscence	0(0%)	3(0.95%)	0.528
Intestinal obstruction	0(0%)	1	0.809
Hospitalization more than 30 days	1(1.33%)	3(0.95%)	0.574
Secondary suture	1(1.33%)	4(1.26%)	0.656
Pre-operative length of stay (LOS)	3.88 ± 2.23	4.74 ± 2.59	0.005^*^
Post-operative LOS	6.03 ± 7.11	8.26 ± 3.90	< 0.001^*^
LOS	9.91 ± 7.70	13.00 ± 4.87	< 0.001^*^
Time to normal activity(weeks)	5.66 ± 3.97	6.92 ± 4.86	0.022^*^
Back to work(weeks)	6.97 ± 3.42	8.08 ± 3.62	0.023^*^
Discomfort symptoms			0.217
Yes	13(17.33%)	76(23.97%)	
No	62(82.67%)	241(76.03%)	

*Note*, Data are presented as the median with the interquartile range (*IQR*, Q1-Q3) or the mean ± SD for continuous variables and as the number and percentage (%) for categorical variables. The p-value was derived by using the Mann–Whitney U test or two-sample t test for continuous variables and by using the Pearson's Chi-square test for categorical variables. ^1^ Conversion from TLH to TAH was one of 75 (1.33%). ^*^p < 0.05

On average, it took significantly less time for patients in the TLH group to return to normal activity than the TAH group (5.66 ± 3.97 weeks vs. 6.92 ± 4.86 weeks, *p* = 0.022). With regards to patients who returned to work, the difference was also significant between the TLH group requiring a shorter time and the TAH group (6.97 ± 3.42 weeks vs. 8.08 ± 3.62 weeks, *p* = 0.0232).

**Fig 2.**
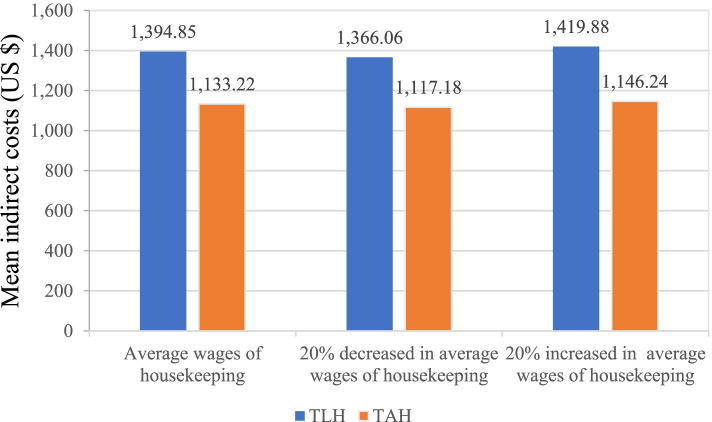
Sensitivity analysis for indirect costs by productivity losses of retired/nonworking patients.

Table 3 presents the costs per case between two patient groups. The average direct medical costs per case of TLH group was significantly higher than that of TAH group (US$2,898.90 vs. US$2,406.88, *p* < 0.001). Combining the statistically indifferent direct non-medical costs, the average direct costs per case of the TLH group significantly higher than that of TAH group (US$2,925.71 vs. US$2,436.14, *p* < 0.001). The average indirect medical costs per case of TLH groups was significantly higher than that of TAH groups (US$1,133.22 vs. US$1,394.85, *p* = 0.006). Time costs of patients were not significantly different between the TAH group and the TAH group (US$887.42 vs. US$1,048.43, *p* = 0.057). Significant difference was observed for the time costs of family members, where the TLH group was lower than the TAH group (US$887.42 vs. US$1048.43, *p* < 0.001). Total costs per case was higher, although not attaining statistical significance, for TLH group compared with TAH group (US$4,058.93 vs. US$3,830.98, *p* = 0.053). Fig. 2 shows that results of sensitivity analysis demonstrated the mean indirect costs was robust.

**Table 3:** Mean costs per case between the TLH and TAH groups (in 2020 US$)

	TLH	TAH	*P*
Direct costs	2,925.71 ± 712.04	2,436.14 ± 557.63	< 0.001^*^
Direct medical costs	2,898.90 ± 708.63	2,406.88 ± 556.1	< 0.001^*^
Direct non-medical costs	26.81 ± 34.13	29.26 ± 47.46	0.615
Indirect costs	1,133.22 ± 637.56	1,394.85 ± 728.53	0.006^*^
Time costs of patients	887.42 ± 602.93	1,048.43 ± 655.72	0.057
Time costs of family members	245.80 ± 111.74	346.42 ± 213.18	< 0.001^*^
Total costs	4,058.93 ± 931.78	3,830.98 ± 949.22	0.053

*Note*, Data are presented as the mean ± *SD*, The p-value was obtained by using the Mann–Whitney U test; ^*^p < 0.05

Table 4 lists the adjusted increment costs and effect and the incremental cost effectiveness ratios. When costs were adjusted for the difference in patient mix by GLM, there were significantly higher total costs for the TLH group versus the TAH group with a mean difference of US$ 256.86. Compared to the TAH group, the TLH group had a lower rate of major surgical complications of 4.53% (95% CI: 4.35% to 4.71%); a shorter time to return to normal activities of 1.27 weeks (95% CI: 1.23 weeks to 1.30 weeks); and, a lower rate of discomfort symptoms of 6.75% (95% CI: 6.45% to 7.05%). The incremental costs for reducing one patient with major surgical complications in the TLH group compared to the TAH group were US$5,669.16 (95% CI: $5,384.76 to $5,955.56). The incremental costs for reducing one patient with postoperative discomfort symptoms in the TLH group compared to the TAH group were US$38.02 (95% CI: $36.35 to $39.68). In addition, the additional costs for reducing the time required until the patient could return to normal activities by one-week, was US$ 202.96 (95% CI: $194.97 to $210.95).

**Table 4:** Cost effectiveness of TLH versus TAH for uterine fibroids

	Adjusted difference (95% CIs)
Increment costs, 2020 US$	256.86 (249.03, 264.69)
Increment effect	
Rate of major surgical complications, %	-4.53 (-4.71, -4.35)
Rate of discomfort symptoms, %	-6.75 (-7.05, -6.45)
Time to normal activity, weeks	-1.27 (-1.30, -1.23)
ICER (Rate of major surgical complications)	-56.69 (-59.55, -53.84)
ICER (Rate of discomfort symptoms)	-38.02 (-39.68, -36.35)
ICER (Time to normal activity)	-202.96 (-210.95, -194.97)

*Note*, *ICER*, incremental cost effectiveness ratio. 95%*CI*, 95% Coefficient Interval

## Discussion

Overall, we found that TLH demonstrated cost effectiveness than TAH from a societal perspective. It was associated with an ICER of $56.69 (95% CI: $53.84 to $59.55) per 1% reduction in major surgical complications and an ICER of $38.02 (95% CI: $36.35 to $39.68) per 1% reduction in discomfort symptoms. Our findings are in line with previous relevant literature which adopted the major complications rate as the primary effect measure [24, 28, 52, 53].

On average, $5,669.16 was invested to prevent one additional major complication when performing a TLH instead of a TAH. Although cost estimate may vary due to differences in perspectives, patient populations, and measurement, most of the published literature favours TLH over TAH regarding overall cost effectiveness [24, 30, 54, 55]. A Dutch randomized trial study found that the TLH is cost effective compared to TAH [52]. Our study reported that the additional costs for reducing one major complication in the TLH group was $5,669.16 as compared to the TAH group. Furthermore, it also found that the higher operative procedure costs for TLH were offset by a shorter LOS for TAH. Further subgroup analysis of the Dutch trial data reported that TLH is cost effective for patients over 70 years of age, but not for patients with a BMI > 35 kg/m^2^ [53]. This study did not capture and analyze, however, the indirect costs of any productivity losses. Furthermore, the TLH and TAH were performed in early endometrial cancer cases, which require more medical resources to treat than uterine fibroids. A systematic review comparing costs and short-term effects between laparoscopic hysterectomy and abdominal hysterectomy, in which twelve prospective trials concerning 2,226 patients in total were reviewed, indicated an ICER of $35,750 to reduce one major complication for laparoscopic hysterectomy compared to abdominal hysterectomy [24]. Noticeably, all these patients had a benign indication for hysterectomy.

According to this threshold of cost to prevent one additional complication to be $10,995 in our study, TLH was a cost-effective alternative to TAH for uterine fibroids in China. Considering that TLH has better long-term outcomes than TAH [23, 24, 26, 53, 54, 56], it is also possible that when incorporating additional longer-term benefits, TLH would become even cost effective than TAH over time.

We have shown that the mean unadjusted total costs of TLH is higher than TAH (US$4,058.93 vs US$3,830.98). This is mainly due to the medical costs in the hospital, which is consistent with the previous literature [24, 38]. Wright et al. also reported that mean total patient costs were US$43,622, US$31,934 and US$38,312 for laparoscopic hysterectomy, abdominal hysterectomy and vaginal hysterectomy, respectively, for benign indications [26]. Our data shows that TLH had higher direct costs but lower indirect costs compared to TAH. The higher direct costs of TLH could be offset by the lower indirect costs to some extent. This is consistent with the previous reports from Sweden and China [13, 38, 57]. The study by Leng et al. from China reported the total costs were $1,065 (direct costs $867, indirect cost $198) in laparoscopic hysterectomy, and $1,050 (direct costs $749, indirect cost $301) in TAH, at 28 days after hysterectomy [38]. The length of follow-up period (12-month in our study vs. 28-day in Leng et al.) and the items considered in the indirect costs (i.e. the productivity losses of both patients and caregivers were considered in our study) may explain the differences in estimated costs. However, a study from Canada found that the total costs of TLH are less than TAH for benign reasons [26], and Barnett et al. reported that total costs of TLH are less than TAH for endometrial cancer patients from a societal prospective [58]. A review showed that direct costs in the laparoscopic hysterectomy group were 6.1% higher than that in the abdominal hysterectomy group ($63,997 vs $60,114), and the indirect costs of laparoscopic hysterectomy were 50% of the indirect costs of abdominal hysterectomy ($1,609 vs $3,139) [24].

With respect to the effect, the TLH group tends to have both lower rate of major surgical complications and lower rate of postoperative discomfort symptoms, and shorter LOS and shorter time back to normal actives compared to TAH. These findings are consistent with the empirical studies conducted elsewhere in the world [13, 22, 25, 26, 59, 60]. Empirical studies from the US [26, 61], the Netherlands [52, 53, 60, 62], the UK [25] and China [63] all report that TLH had lower but insignificant complications rates compared to TAH. In addition, several studies from the US [41] and from Germany [64] report that TLH was associated with significantly lower overall rate of intraoperative complications and minor postoperative complications than TAH.

Regarding the LOS and postoperative hospitalization period, consistent with the literature, both indicators are statistically significantly shorter in the TLH group [23, 41, 65]. However, in our study, the LOS and postoperative LOS are longer compared to other studies conducted in developed countries. The fee-for-service payment system adopted in China may be the main explanation for the difference. Health care providers have an incentive to keep patients in hospital longer [66]. The difference in medical technology levels between China and other developed countries could be another possible reason.

The main strength of our study is that it presents the first analysis of the incremental cost effectiveness of TAH and TLH for patients with uterine fibroids in mainland China, not to mention in Western China. In addition, this study assessed not only the direct costs, but also the productivity losses of patients and caregivers. Moreover, we followed up 12-month period to obtain the data of costs and outcomes. The findings provide useful information for researchers to conduct further cost effectiveness analysis based on prospective study which can provide stronger and more evidence, in China. In addition, the data may be useful for Chinses health care policy-makers and medical insurance payers to make related health care decision.

A few limitations should be considered when interpreting the results of this study. Firstly, this is a retrospective study and our patients are from a single hospital, thus a sample selection issue may exist and potentially limit the generalization of our findings. Secondly, using all major surgical complications as the primary measure of effectiveness, assumes that each type of major surgical complication is fundamentally equivalent to each other. Thirdly, this study only followed patients for 12 months. The long-run quality of life effects for the different patient groups was not studied. Fourthly, by the time this study was conducted, new surgical procedures, such as robotic-assisted laparoscopic surgery for hysterectomy, have flourished in the developed countries [26, 67–69]. However, to date, such new techniques have not been widely adopted in China.

## Conclusions

In conclusion, according to our study, TLH is cost effective compared to TAH, based on a threshold calculated at $10,995, to prevent one additional complication in China. Further randomized, prospective studies would be an ideal way to further explore the incremental costs and long-run health-related quality of life of different hysterectomy procedures in China.

## Data Availability

The data presented in current study are available on reasonable request from the corresponding author.

## Abbreviations

TAH: Total abdominal hysterectomy
TLH: Total laparoscopic hysterectomy
ICER: Incremental cost effectiveness ratio
LOS: Length-of-hospital stay
QOL: Quality of life
QALYs: Quality-adjusted life years
GDP: Gross domestic product
BMI: Body mass index
EBL: Estimated blood loss
GLM: General liner models
SD: Standard deviation
NCMS: New Cooperative Medical Scheme
URBMI: Urban Resident Basic Medical Insurance
UEBMI: Urban Employee Basic Medical Insurance
95%CI: 95% Coefficient Interval
